# Planning for complex inferior vena cava filter retrievals: the implementation and effectiveness of 3D printed models

**DOI:** 10.1186/s41205-024-00226-x

**Published:** 2024-10-05

**Authors:** Joonhyuk Lee, Frank J. Rybicki, Prashanth Ravi, Seetharam C. Chadalavada

**Affiliations:** 1 Department of Radiology, University of Cincinnati Medical Center, Cincinnati, OH 45219 USA; 2 Department of Radiology, University of Arizona College of Medicine, Phoenix, AZ 85004 USA

**Keywords:** 3D printing, Interventional Radiology, Additive Manufacturing, Rapid Prototyping, Anatomic Model, IVC Filter, Thromboembolic Disease

## Abstract

**Background:**

Inferior vena cava filter (IVC) retrieval is most often routine but can be challenging with high morbidity in complex cases, especially those with an extended dwelling time. While risk of morbidity in complex retrievals is decreased with advanced filter retrieval techniques, deciding when and which to use these requires detailed pre-procedural planning. The purpose of our study was to evaluate patient-specific 3D printed anatomic IVC filter models for aiding complex IVC filter retrievals.

**Methods:**

All IVC filter retrieval patients between June 2021 and September 2022 at one academic medical hospital were prospectively screened. Nine met criteria for complex retrieval, and their CT images were used to 3D print patient-specific IVC and filter models. Models were used in pre-procedural planning and clinical utility was assessed using the Anatomic Model Utility Likert Questionnaire and estimations of the procedural and fluoroscopy time saved.

**Results:**

The usage of 3D printed models in pre-procedural planning had high clinical utility based on the Likert questionnaire (Anatomic Model Utility Points 366.7 ± 103.1). Using a model significantly increased confidence in planning (*p* = 0.03) and modified the treatment plan in seven cases. It also led to cost-efficient use of resources in the procedure suite with estimated reduction in procedure and fluoroscopy time of 29.0 [20.3] (*p* = 0.003) and 10.2 [6.7] (*p* = 0.002) minutes, respectively.

**Conclusion:**

3D printed anatomic models for patients who require complex IVC filter retrieval demonstrated Likert-based high clinical utility and led to estimated reductions of procedural and fluoroscopy time.

## Background

Inferior vena cava (IVC) filter placement has guideline support [1] for patients with acute venous thrombosis and a contraindication for anticoagulation. Retrieval is indicated when the risk of venous thromboembolism (VTE) is resolved, or if anticoagulation is initiated and the complications outweigh the benefits of the filter. Removal of filters with a long dwell time can be challenging and are subject to failed retrievals, procedural complications, and higher morbidity [1, 2].

To our knowledge, there is not yet a widely recognized or standardized definition for a ‘complex’ retrieval patient. For this project, a retrieval was considered complex if any of the following criteria were met: extended dwell length (> 7 months), IVC thrombus, fibrin sheath presence, IVC wall penetration by the filter, filter tilt greater than 15 degrees from the craniocaudal axis of the IVC, or filter fracture [3, 4].

Complication risk is lowered with interventional radiologist experience and advanced techniques, such as use of forceps, laser sheath, or dual access to name a few. However, these are inherently riskier [4–7]. The purpose of our study was to evaluate patient-specific 3D printed anatomic IVC filter models for aiding complex IVC filter retrievals.

## Methods

### Patients

This study underwent IRB approval by the human research committee at one, urban, adult academic medical center. All patients who had an IVC filter removal within the study period (June 2021– September 2022) were prospectively evaluated to determine if the procedure met the criteria for complex, after which written informed consent was obtained.

### Pre-procedural planning and 3D printing

All patients had a pre-procedural clinic visit and according to our institutional IVC filter retrieval protocol, a pre-operative CT scan of the abdomen and pelvis was obtained with and without contrast. Image acquisition used 64 or more detector rows; the tube voltage and tube current were set by automated software. Axial slices were reconstructed with a soft tissue kernel at 1.0–1.5 mm slice thickness.

Pre-procedural CT images were reviewed by a diagnostic radiologist with 30 + years of experience with 3D visualization in the Digital Imaging and Communications in Medicine (DICOM) space using multiplanar reformatted images and volume rendering.

The IVC filter and relevant vascular structures, such as the inferior vena cava or lumbar veins, were segmented using Materialise Mimics (Materialise, Leuven, Belgium). Computer-Aided Design (CAD) was used to manipulate the surface mesh using Materialise 3-Matic (Materialise, Leuven, Belgium). The surface mesh was overlaid onto the DICOM data, and one radiologist verified the accuracy of the part before 3D printing using desktop inverted vat polymerization (VP, Form 3B, Formlabs, Somerville, MA, USA) or full color material jetting (MJT, Stratasys J5, Stratasys, Eden Prairie, MN, USA). For each patient, the 3D printed anatomic model was provided to one interventional radiologist who performed all procedures.

### IVC filter retrieval

Venous access was obtained using ultrasound, first in the jugular vein and additional points as needed in the femoral veins. Cavography was used to assess for in-situ filter thrombus, venous anatomy, and flow. When possible, standard retrieval using endovascular snare and sheath was attempted. If significant resistance was met, or if pre-procedurally determined to be ineffective based on the presence of one or more complex risk factors present on imaging or 3DP models, advanced retrieval techniques were used such as the loop snare, forceps, or laser excimer sheath (CavaClear, Philips, Amsterdam, Netherlands) [8]. Post-retrieval cavography determined the need for angioplasty and stent placement based on residual flow-limiting stenosis.

### Post-procedural follow-up

Patients were observed for complications. The 4 patients who required stenting were kept for overnight observation and had clinical and CT follow-up within 2 months. All patients underwent 6 months post-procedure chart review.

### Assessment of 3D printing

The treatment plan for filter retrieval was determined by an interventional radiologist with 20 + years’ experience. Initial planning was based on CT imaging and 3D visualization of the case using all available DICOM data sets. That interventional radiologist then used the anatomic model as an additional visual aid to confirm or adjust their treatment plan as needed (Table 1). The confidence in the treatment plan before and after using the 3D printed model was benchmarked. Alongside, the five-point scale Likert questions [9] from the American College of Radiology – Radiological Society of North America 3D Printing Registry were answered by the same interventional radiologist using the imaging as a baseline (Table 2). The Likert results were converted to Anatomic Model Utility Points [9]. After the procedure, the procedure and fluoroscopy time saved due to use of the 3D printed model were estimated by the interventional radiologist.

**Table 1:** Procedural Summary Upon Use of an Anatomic Model. Anatomic model driven adjustments in pre-procedural planning are described. The anatomic models were especially useful in planning whether more vascular access points were required and which retrieval technique to use, thereby saving time in the operative suite

Patient	Alteration in approach upon 3DP model use	Access points	Filter retrieval technique used
1	- Adjusted number of access points - Decided which filter to target first - Assessed filter integrity and confirmed fractured components	- Right internal jugular - Right common femoral	- Forceps
2	- Guided choice of retrieval technique - Helped determine time spent attempting simple retrieval techniques before transitioning to advanced	- Right internal jugular	- Sheath and snare - Forceps
3	- No alterations	- Right internal jugular	- Sheath and snare
4	- Guided choice of retrieval technique - Helped determine time spent attempting simple retrieval techniques before transitioning to advanced	- Right internal jugular	- Sheath and snare - Forceps
5	- Adjusted number of access points - Helped plan angioplasty and stenting	- Right internal jugular - Bilateral femoral	- Forceps
6	- Adjusted number of access points - Helped plan angioplasty and stenting	- Right internal jugular - Bilateral femoral	- Forceps
7	- Adjusted number of access points - Helped plan angioplasty and stenting	- Right internal jugular - Bilateral femoral	- Forceps
8	- Adjusted number of access points - Assessed filter integrity and confirmed fractured components	- Right internal jugular - Right common femoral	- Forceps - Laser excimer sheath
9	- No alterations	- Right internal jugular	- Sheath and snare - Laser excimer sheath

**Table 2:** Likert Questionnaire Summary. Likert questions reported using a 5-point scale; 1 = strongly disagree, 2 = disagree, 3 = neutral, 4 = agree, 5 = strongly agree. Mean and Standard Deviations are reported for nine patients who underwent 3D printing for complex IVC filter retrieval. Specialists’ responses were converted to anatomic model utility points (AMUPs). Responses of “strongly disagree”, “disagree”, and “neutral” were assigned 0 AMUP points. Responses to pre-procedural confidence were assigned negative points to effectively subtract the impact of the anatomic model post- versus pre-procedure. The maximum AMUP for each patient was 500

Likert Questions from 3D Printing Registry [9]	Mean Likert Score	SD of Likert Score	Response and Conversion to AMUPs				
			Strongly disagree	Disagree	Neutral	Agree	Strongly Agree
**The 3D printed model was easy to use**	4.9/5	0.3	0	0	0	25	50
**Before using the 3D printed model**,** I was confident in the treatment plan**	3.9/5	0.8	0	0	0	-50	-100
**After using the 3D printed model**,** I was confident in the treatment plan**	4.9/5	0.3	0	0	0	50	100
**As a result of using the 3D printed model**,** the treatment plan was altered or refined**	4.2/5	0.8	0	0	0	50	100
**Use of the 3D printed model was important in this case**	4.4/5	0.5	0	0	0	50	100
**The quality of the 3D printed model was adequate**	4.9/5	0.3	0	0	0	50	100
**Use of the 3D printed model was compatible with other aspects of my approach this case**	4.9/5	0.3	0	0	0	25	50

### Statistics

Statistical comparisons evaluated confidence in treatment plan before and after using the 3D printed anatomic model with a Wilcoxon signed-rank test. For the procedure and fluoroscopy time saved, the distribution of the recorded times vs. the recorded times plus the estimated times saved were compared. The distribution was determined to be normal or non-normal using a Shapiro-Wilk test. Comparison statistics were then performed respectively using a two-tailed Student’s t-test assuming unequal group variance or a Mann-Whitney U test. Significance levels were defined at 0.05.

## Results

### Patients

Among the 41 patients who underwent IVC filter retrieval within the study period, nine patients (6 male; mean [SD] age of 60.2 [14.4] years) met the criteria for complex (Table 3), and all signed written informed consent. Eight of the nine patients had the filter placed at an outside institution. The mean [SD] dwell time was 8.4 [7.0] years; range 0.2–19.3 years. Eight patients had extended dwell time (> 7 months) [6].

**Table 3:** Patient Data Summary. Patients who underwent 3D printing and complex IVC filter retrieval. Patient 9 was 3D printed using material jetting (MTJ) to delineate additional anatomy that was important for removal. This required a significantly longer print time and material volume than vat polymerization (VP) 3D printing used for the other patients. Anatomic modeling utility points (AMUPs) are reported for each patient based on the scoring system in Table 2

Patient	Filter Type	Reason for Complex Classification	Filter Dwell-Time (years)	AMUPs	Additional Parts in Model	Clinician Time (min)	Non-Clinician Time (min)	3D Printer (Technology)	Print Time (min)	Post-Print Processing Time (min)	Materials Used	Material Volume (cc)
1	Double-stacked Cook Gunther Tulip Filters	Extended dwell, tip embedded, significant tilt, thrombus, prior attempt and failure of retrieval at outside hospital, poorly accessible	15.7	500	-	60	330	Form3B (VP)	150	60	Clear Resin	5.6
2	Bard Eclipse/G2X filter	Extended dwell, fibrin sheath, leg extension into renal veins	12.7	350	-	30	150	Form3B (VP)	105	60	Clear Resin	4.5
3	Cook Celect	Extended dwell, fibrin sheath, extravascular struts	12.7	300	-	15	105	Form3B (VP)	225	60	Clear Resin	6.7
4	Denali	Tip adherence to wall, previously failed sheath/snare retrieval	0.2	200	-	30	210	Form3B (VP)	120	90	Clear Resin	4.2
5	Denali	Extended dwell, contracted morphology of IVC filter with severe stenosis of the vessel and extensive collaterals	1.2	450	-	30	210	Form3B (VP)	105	60	Clear Resin	3.5
6	Option Elite	Extended dwell, thrombus formation below the filter extending to but not into the iliac veins, extended dwell, poorly accessible	4.5	450		30	180	Form3B (VP)	120	90	Clear Resin	2.8
7	Denali	Extended dwell, thrombus formation, previously aborted attempt at outside hospital	1.3	450	-	30	105	Form3B (VP)	120	60	Clear Resin	2.2
8	Trapease	Extended dwell, embedded strut, fractured filter, extracaval penetration of strut, significant tilt, possible thrombus, poor accessibility	19.3	350	-	15	105	Form3B (VP)	180	60	Clear Resin	9.3
9	Denali	Extended dwell, fibrin sheath suspected, tip embedded in lumbar vein, probable fractured strut	5.6	250	IVC; lumbar vein	30	255	Stratasys J5 (MJT)	585	60	MED610; DraftWhite; VeroCyan-V; VeroMagenta-V; VeroYellow-V; SUP710	125.0

### Pre-procedural planning and 3D printing

**Fig. 1:**
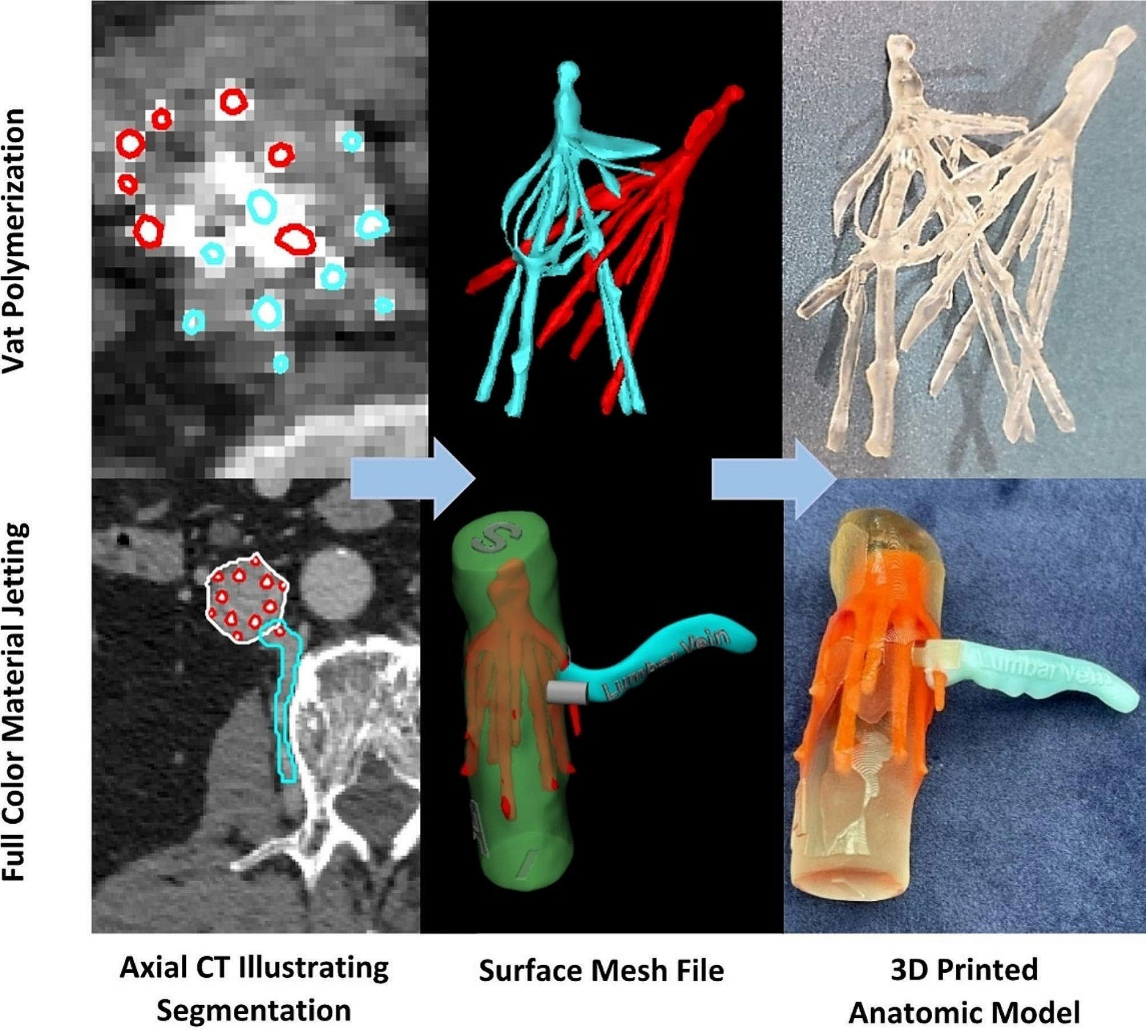
Rapid Prototyping of Patient-specific IVC Filter Models. (Top) 3D printing workflow illustrated for a double-stacked Cook-Gunther Tulip filters (Beckton Dickinson, Franklin Lakes, NJ, USA) additionally complicated by extended dwell and prior failure in retrieval at an outside institution. The anatomic model was 3D printed with inverted vat polymerization and clear resin. (Bottom) 3D printing workflow illustrated for a Denali filter complicated by a 9-year dwell-time with penetrating strut through the lumbar vein and possible fracture. The anatomic model was 3D printed in color using material jetting to distinguish position of the filter (orange) with relation to the IVC (clear) and lumbar vein (blue)

The nine patients had 3D printed models constructed using either vat polymerization (*n* = 8 of 9) or material jetting technology (*n* = 1 of 9) (Figure 1). 3D visualization images were sent to the hospital picture archiving and communication system for review by the interventional radiologist. For the eight vat polymerization patients, the mean (SD) 3D printing time was 140.6 (42.4) minutes (Table 3). The single material jetting patient is indexed (Table 3).

### IVC filter retrieval

The anatomic model was used pre-procedure to help determine the number of access sites (Table 1). Patients 2, 3, 4, and 9 had single right internal jugular (RIJ) access; Patients 1 and 8 required a dual access (RIJ + right common femoral vein); Patients 5, 6, and 7 required triple access (RIJ + bilateral common femoral veins). For all patients, except Patients 3 and 9, retrieval used forceps [4]. Patient 3 required only a snare for the IVC filter retrieval. For patients 8 and 9, a laser sheath was used due to severe endothelization [8]. Patients 5, 6, 7, and 8 required IVC and bilateral common iliac vein stenting. Patients 6 and 7 also required stenting of the left external iliac vein. All patients with stenting had satisfactory flow post placement. No intra-procedural complications were noted.

The only peri-procedural complication was readmission of Patient 8 several days after filter retrieval. She presented with a small right groin hematoma at the incision site and segmental pulmonary embolism without right heart strain. Complications were managed medically with discharge the next day in a stable condition.

### Post-procedural follow-up

Status-post removal of the filter, all patients were on anticoagulation. Patient 1 was also followed up clinically because there was a small retained filter strut which required monitoring and confirmed to be ossified and extracaval. None of the patients had an additional complication within 6 months. All three patients (5, 6, and 8), who were initially symptomatic reported significantly improved activity levels and reduced lower extremity swelling.

### Assessment of 3D printing

Confidence in the treatment plan was significantly improved after utilizing the 3D printed anatomic model (3.9/5 vs. 4.9/5 respectively, *p* = 0.03). The mean (SD) of the AMUP was 366.7 (103.1) (Table 2); maximum score of 500. There were significant decreases in the estimated procedural time, 29.0 [20.3] minutes (*p* = 0.003), and the estimated fluoroscopy time, 10.2 [6.7] minutes (*p* = 0.002). The data followed a normal distribution. There were no complications attributed to the 3D printed anatomic model.

Anatomic models led to altered treatment approach in all patients except Patients 3 and 9 (Table 1). For example, Patient 1 (intertwined double IVC filter; 16-year dwell-time) was changed from a singular jugular to a combined jugular and femoral venous access. This patient also had an ossified fibrin sheath containing an extra-caval filter strut that precluded removal in its entirety; only a small fragment remained in the patient [11]. The anatomic model (only the IVC filter, printed with VP) contributed to the pre-procedural decision to not attempt removal of this small fragment. The model was also highly useful to match the extracted components and identify the expected location of the ossified strut which increased confidence that the outcome matched expectations from intra-procedure fluoroscopy.

The anatomic models were highly impactful for hospital workflow including the interventional radiology lab time allocated for the case, resource use such as a laser excimer sheath [8] that is shared with other departments, the location and size of the access points and the order in which they are used, and the decision to perform the entire complex filter retrieval as a single procedure or over more than one day. In addition, they guided the choice of filter retrieval technique.

For Patient 9, the more complicated MTJ 3D print was requested because the patient needed and requested removal, despite two failed IVC removal attempts at outside institutions that were attributable to the relationship between the filter, the IVC, and draining lumbar veins. For each of these parts, the segmentation, CAD, and 3D printing in distinct colors provided the geometric (intra- versus extra-vascular) relationship of the fracture fragments for safe removal.

## Discussion

The high utility of 3D printed anatomic models for patients who require complex IVC filter retrieval supports its clinical use. Anatomic models improved the confidence of the interventional radiologist and enabled efficient use of hospital resources. Considering an estimate of $100 USD per minute in the interventional radiology suite, the potential savings was approximately $2900 / per patient [10].

Because IVC filters are small and have high utility in a single material and color, 3D printing the filter is amenable to an efficient workflow and lower cost with one day turn-around time. More complex and resource-intensive printing should be further studied when the IVC itself, tilt angle, and or extracaval stent parts are required for planning. For example, material jetting requires a two day turn-around because the anatomic model requires additional segmentation and CAD, and the expected print time is greater than 8 hours.

With regards to current literature, the use of 3D printing in interventional radiology is sparse, especially in comparison to interventional cardiology [12], promoting research of further avenues of application in the field. To date, no other studies have used personalized 3D printed anatomic models to guide pre-procedural planning for IVC filter retrieval. The only other similar study printed IVC phantoms to test filter placement techniques; however, this study did not involve active patient care or print filter models [13]. The high clinical utility of patient-specific models in our study help demonstrates a new opportunity for 3D printing to be useful in interventional radiology.

Limitations of this study include the small sample size. However, the patient cohort was adequate to show that treatment plan was significantly improved. There was also a diversity of complicated IVC filter issues such as multiplicity of filters, orientation, degree of fracture, or extravascular involvement. Additionally, only one patient underwent material jetting. However, this case illustrated that draining veins can be added to the anatomic model to allow for better characterization of complex features. In particular, it was useful to characterize the position of the strut penetrating the lumbar vein to help confirm whether removal via laser sheath was feasible.

A second limitation is that the physical properties of the 3D printed stent do not allow the interventional radiologist to test the mechanics of how the parts may deform during removal. Future research can iterate among existing and newer materials for desktop printing utility for the interventional radiologists.

## Conclusions

While clinical 3D printing should likely not exist as a stand-alone tool for pre-procedural guidance in complex IVC filter retrieval, it presents itself as a powerful tool to improve visualization and confirm or even alter the operative plan. Anecdotally, the operating interventional radiologist found the filter to be especially useful in cases with significant dwell-time, a known risk-factor for filter failure. In this small study, 3D printed patient-specific models had a positive impact on patient care and improved the confidence of the interventional radiologist who performed the procedure. Estimations suggest a significant reduction in procedural and fluoroscopy time, as well as more efficient use of hospital resources though such reductions. The low volume of material used along with the rapid speed of 3D printing using desktop 3D printing indicate the high potential for this approach to be adopted at other centers and transform pre-procedural planning for complex IVC filter retrieval cases.

## Data Availability

The datasets used and/or analyzed during the current study are available from the corresponding author on reasonable request.

## Abbreviations

AMUP: Anatomic Model Utility Points
CAD: Computer-Aided Design
DICOM: Digital Imaging and Communications in Medicine
IVC: Inferior vena cava
MJT: Material jetting
RIJ: Right internal jugular vein
VP: Vat polymerization
